# HPV insertional pattern as a personalized tumor marker for the optimized tumor diagnosis and follow-up of patients with HPV-associated carcinomas: a case report

**DOI:** 10.1186/s12885-019-5447-1

**Published:** 2019-03-28

**Authors:** Alexandre Harlé, Julie Guillet, Jacques Thomas, Jessica Demange, Gilles Dolivet, Didier Peiffert, Agnès Leroux, Xavier Sastre-Garau

**Affiliations:** 1 Université de Lorraine, Nancy, France; 2 CNRS, UMR, 7039 CRAN, Nancy, France; 3 Service de Biopathologie, Institut de Cancérologie de Lorraine, Vandoeuvre-lès-Nancy, France; 4 Département de chirurgie oncologique, Institut de Cancérologie de Lorraine, Vandoeuvre-lès-Nancy, France; 5 Département de radiothérapie, Institut de Cancérologie de Lorraine, Vandoeuvre-lès-Nancy, France

**Keywords:** HPV, Tumor biomarkers, Anal carcinoma, Head & neck carcinoma

## Abstract

**Background:**

In clinical oncology, only a few applications have been developed using HPV as a personalized tumor marker, a lack most probably related to the limited information obtained by the classical Polymerase Chain Reaction (PCR) approach. To overcome this limitation, we have recently developed the capture-based Next-Generation Sequencing (NGS) “CaptHPV” assay, designed to provide an extensive and comprehensive molecular characterization of HPV DNA sequences associated with neoplasias, ie the sequence of the viral genome (245 genotypes), its physical state, viral load, integration site and genomic alterations at integration locus. These data correspond to highly specific tumor markers that can be used to improve diagnosis and patient’s follow-up.

**Case presentation:**

We report here a case that is a straightforward and practical illustration of the power of the CaptHPV method. A patient developed successively a carcinoma of the anal canal and of the tongue. The two tumors were squamous cell carcinoma, found associated with HPV16 using PCR. In order to document a possible metastasis to the tongue from the anal cancer, we performed CaptHPV analysis on the two tumors. The analysis of the anal carcinoma found 55 viral/human hybrid reads allowing the identification of the HPV16 DNA integration in the 4q25 chromosomal band locus with a 178,808 bp deletion in the cell genome. Molecular analysis of the tongue tumor disclosed 6110 reads of HPV16, with a viral pattern strictly identical to that of the anal tumor. A total of 131 hybrid reads between HPV16 and the cell genome were found, corresponding exactly to the same locus of integration of viral DNA at the 4q25 site. The 178,808 bp genomic deletion was also found in the lingual tumor. The exact identity of HPV insertional signatures in the two tumors, demonstrates unambiguously that the tongue tumor derived from the anal cancer whereas neither histological immunophenotyping nor classical viral analysis using PCR could allow a definitive diagnosis.

**Conclusion:**

Our observation indicates that the establishment of a detailed cartography of HPV DNA sequences in a tumor specimen provides crucial information for the design of specific biomarkers that can be used for diagnostic, prognostic or predictive purposes.

## Background

Specific genotypes of human papillomaviruses (HPV) are associated with a large number of carcinomas developed in the mucosa of the ano-genital and of the head and neck tracts. The prevalence of HPV is different according to tumor localization and has been described in 96% in cervical [1], 88% in anal [2], 22.5% in vulvar [3], 42% in penile [4] and 40% in head & neck [5] carcinomas. A large heterogeneity in viral genotypes exists and histo-virological analyses have shown that three HPV groups could be identified according to their oncogenic properties: Low-risk HPVs (e.g. HPV6, 11, 42, 43, 44...) present in low grade intraepithelial neoplasias but rare in invasive cancers, intermediate risk HPVs (e.g. HPV31, 33, 35, 51, 52, 58...) more prevalent in intraepithelial neoplasia than in invasive cancers and high-risk HPVs (HPV16, 18, 45, 56), more prevalent in invasive cancers than in intra-epithelial neoplasias [6]. The physical state of the viral genomes is different according to the type of lesions. In intraepithelial neoplasias, viral double stranded DNA multiplies as free molecules in the nucleus of infected cells whereas, in the majority of invasive cancers, at least part of the viral genome is integrated into the tumor cell genome [7]. This leads to genomic alterations often characterized by a co-amplification of viral and flanking cellular sequences [8]. Many studies have focused on the oncogenic properties of HPVs and the use of HPV detection for the screening and characterization of preinvasive neoplasia [9, 10]. In contrast, in clinical oncology, a few applications have been developed using HPV as a tumor marker. HPV has been recognized as a biological prognostic factor in cervical [1] and in head & neck cancers [11] and the determination of HPV status is recommended in this later tumor type [12]. More recent studies focused on the detection of circulating HPV DNA as a predictive marker of response to therapy [13, 14].

This lack in developments of clinical applications based on HPV as a tumor marker in the follow-up of patients with HPV-associated invasive carcinoma is most probably related in part to the limited information obtained by the Polymerase Chain Reaction (PCR) approach, frequently used to determine the HPV genotype. This method is very sensitive but provides a qualitative result, meaning the presence or absence of HPV DNA, based on the detection of a short DNA sequence related to a specific or to a limited number of HPV genotypes. Information about the exact sequence of the HPV genome, its physical state - episomal or integrated-, the integration site and the presence of genomic change at the insertion site, such as amplification or deletion, are missing. Thus all features that constitute personalized biomarkers specific for each tumor are lacking. To overcome this limitation, we have recently developed the capture-based Next-Generation Sequencing (NGS) “CaptHPV” assay. The CaptHPV assay has been designed to provide an extensive and comprehensive molecular characterization of HPV DNA sequences associated with neoplasias. CaptHPV allows, in a single experiment, the identification of the physical state, viral load, insertion site and presence of genomic alterations in the specimen for 245 different HPV genotypes. This approach, validated on a retrospective series of 72 cases of invasive carcinoma of the uterine cervix [15], revealed to be robust and appropriate for use in clinical practice to obtain personalized molecular signature for each tumor.

We report here a patient case who developed two HPV-associated tumors, first in the anal canal then in the tongue. Classical histological and virological analyses could not permit to conclude whether this corresponded to the development of independent tumors, or to the secondary spread to the tongue of the primary anal tumor. Using CaptHPV, we were able to show that the two tumors harbored identical HPV insertional signatures, demonstrating that the tongue tumor corresponded to the metastatic spread of the anal carcinoma. We think that, in the near future, this innovative approach will be of large use to determine a detailed cartography of the viral sequences that characterize any HPV-associated carcinoma, providing specific tumor markers useful for patients’ follow-up.

## Case report

**Fig. 1:**
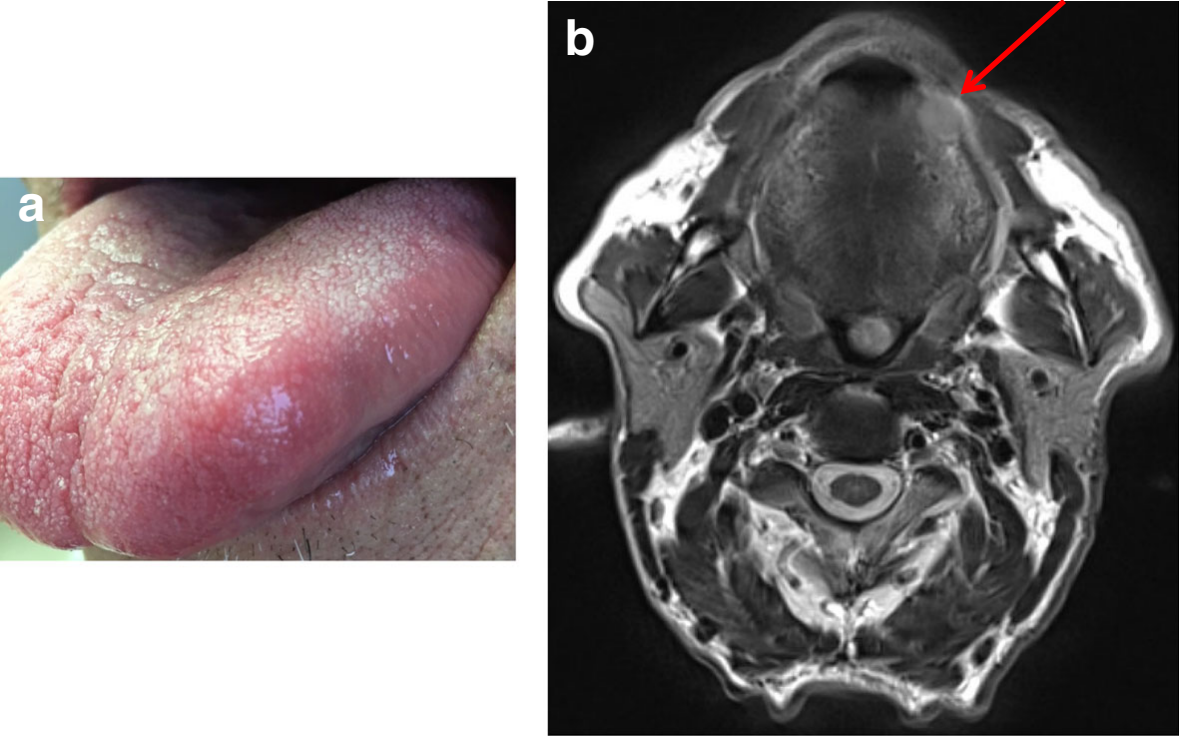
Lingual tumor localized on the left side of the tongue: clinical aspect (**a**) and MRI imaging (**b**) showing the 11 × 11 mm tumor mass

**Fig. 2:**
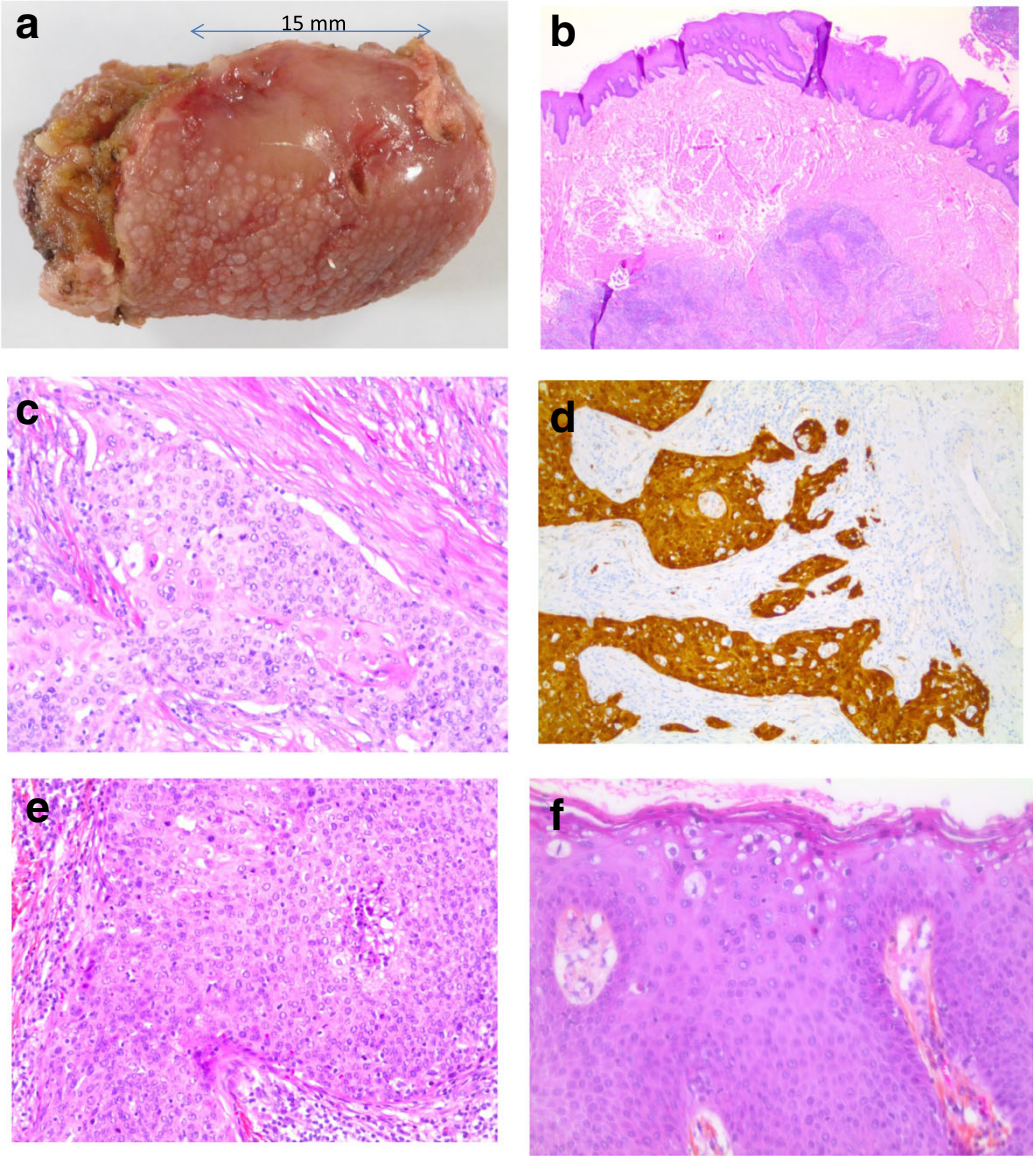
Morphological aspects of the lingual (**a** to **d**) and anal (**e**, **f**) tumors. Surgical resection showing the tumor at the left margin of the tongue (**a**). Microscopic examination showing the submucosal localization of the lingual tumor (**b**). Histological aspect of keratinizing squamous cell carcinoma (**c**) with strong p16 expression (**d**). Histological aspect of squamous cell carcinoma of the anal canal (e) and of the condylomatous lesion in the epithelium distant from the tumor (f)

In January, 2018, a 58 years old male patient was hospitalized for a tumor of the tongue (Fig. 1a). As comorbidity factors, a gastric ulcer (1983), vein thrombosis (2016), smoking and alcohol abuse stopped in 2015, were documented. In 2017, the patient was treated by radio-chemotherapy for an invasive carcinoma of the anal canal, stage T3NxM0. At examination, the lingual tumor was localized on the submucosal part of the left side of the tongue and measured 11 × 11 mm at MRI (Fig. 1b). The patient benefited from a surgical removal of the tumor (Fig. 2a). The histological analysis of the specimen showed that the bulk of the tumor was localized in the tongue muscle, the upper part of the lesion remaining at 0.5 mm of the basal membrane (Fig. 2b). The epithelium was normal, without ulceration or intra-epithelial neoplasia. At higher magnification, the tumor corresponded to a squamous cell carcinoma (SCC), keratinizing (Fig. 2c). The stroma presented moderate lymphocytic infiltration. No vascular or peri-neural invasion was seen. Immunophenotyping revealed a strong p16 labeling (100% of the cells) (Fig. 2d). The tumor was well limited in the periphery and the surgical margins were clear.

**Fig. 3:**
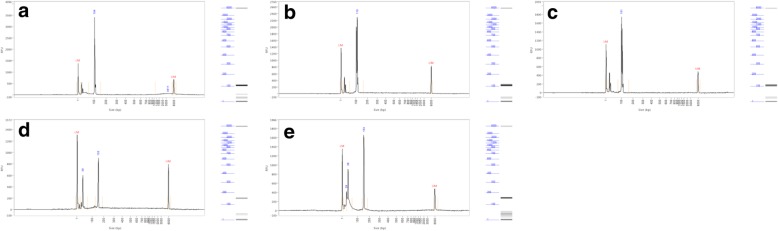
PCR migration profiles using Fragment analyzer. HPV16 amplification have been found in both lingual tumor and anal carcinoma with relative fluorescence unit (RFU) of 3411, 2298 and 1740 for lingual tumor (**a**), anal carcinoma (**b**) and positive control (**c**) respectively and amplification of HPV consensus has only been found in anal carcinoma relative fluorescence with RFU of 901 and 1654 for anal carcinoma (**d**) and positive control (e) respectively. LM: Lower Marker; UM: Upper Marker

**Fig. 4:**
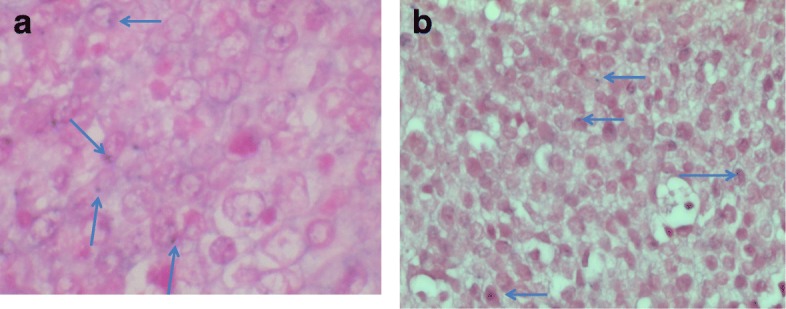
Positive signal characterized by a dot close to the nuclear membrane (arrows) of lingual (**a**) or anal (**b**) tumor cells

Considering the previous diagnosis of carcinoma of the anal canal, the histological features of the two tumors were compared and complementary virological analyses were performed in order to document a possible metastasis to the tongue from the original primary anal tumor. Histologically, anal and lingual tumors corresponded to bona fide SCCs (Fig. 2c, e). As compared to the lingual tumor, the keratinization in the anal carcinoma was less pronounced and a poorly differentiated component somewhat reminiscent of a basaloid carcinoma was also present, exhibiting necrotic foci. At the upper part of this anal tumor specimen, there was an ulceration edged by lateral extension of high grade intra-epithelial neoplasia. In addition, on the epithelium, distant from the ulceration, there were condylomatous changes characterized by the presence of irregular nuclei, monocellular dyskeratosis and koilocytes at the surface layer of the epithelium (Fig. 2f). Immunohistochemistry showed a strong p16 expression (100% of the cells), also observed in the intra-epithelial and condylomatous lesions. HPV detection was performed by PCR using primers specific for HPV16 and HPV18, as well as the consensus GP5/GP6 primers. This analysis found HPV16 DNA in the two tumor specimens (Fig. 3) and consensus HPV sequences in the anal carcinoma specimen. In situ hybridization was performed using the INFORM HPVIII Family 16 probe (Ventana Medical Systems Tucson, AZ, USA) detecting HPV16, 18, 31, 33, 35, 45, 52, 56, 58, 66 genotypes. This analysis showed the presence of scattered dot signals, located close to the nucleus membrane of the tumor cells in anal and lingual carcinoma (Fig. 4), corresponding to the presence of integrated HPV DNA sequences. No signal was found in the surrounding tissue or in the epithelium. The condylomatous lesion in the anal specimen was negative.

**Fig. 5:**
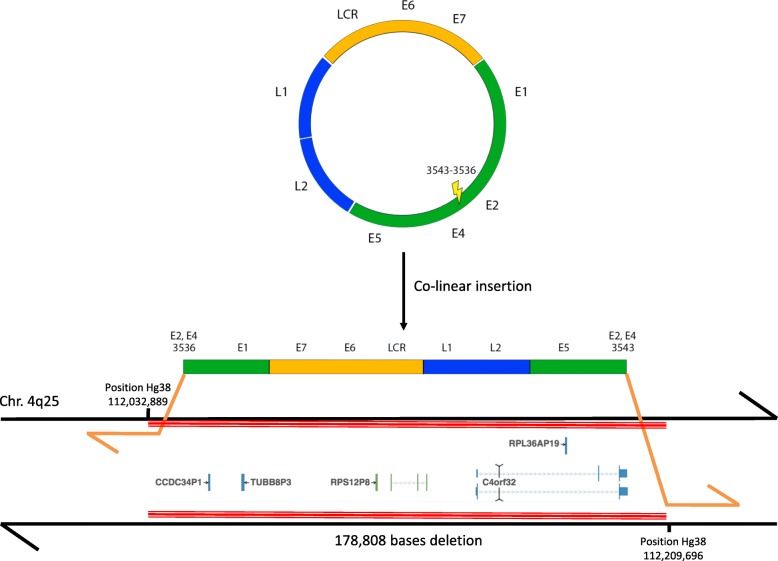
Stable HPV16 integration in the tumor cell genome with chromosome deletion. Mapping of the two viral/cell junctions in a co-linear (2J-COL) pattern is shown. The coordinates of the human (junction to chromosome, *hg38* reference) and viral (junction to junction) are indicated, as well as the chromosomal locus of insertion. HPV16 insertion is associated with the deletion of 178,808 bp in the cell genome, including *FAM241A* and four pseudogenes

The two tumors presented thus substantial histological and viral similarities but these observations could not permit to conclude metastasis originating from anal tumor since similar tumors associated with the same HPV genotype could be developed independently in the two organs. Therefore, we decided to further analyze these two tumors using the CaptHPV assay in separate experiments, in order to see if specific viral signature could help to make the diagnosis more precise. Using this assay, HPV53 and HPV16 genomes were found in the anal carcinoma (5258 and 75,990 reads, respectively, out of 111,644 reads including 3 human one-copy housekeeping genes as a control for the CaptHPV method). Whereas HPV53 DNA sequences corresponded to the full length viral genome, most probably related to viral episomes, HPV16 DNA showed a breakpoint on the E2/E4 ORFs with a deletion of 6 nucleotides. The LCR, E6 E7 L1 and L2 ORFs were conserved. The identified integration mechanism of HPV16 in the tumor cell genome was that of two junctions in a co-linear pattern (2J-Col). Analysis found 55 viral/human hybrid reads allowing the identification of the HPV16 DNA integration in the 4q25 chromosomal band locus with a 178,808 bp deletion in the cell genome corresponding to the deletion of *C4orf32* (*FAM241A*) gene and CC*DC34P1, RPS12P8, RPL36AP19 and TUBB8P3* pseudogenes (Fig. 5). In the carcinoma of the tongue, molecular analysis disclosed 6110 reads of HPV16, without evidence of HPV53 DNA. The viral pattern was strictly identical to that observed in the anal tumor: conservation of the LCR, E6 E7 L1 and L2 ORFs and breakpoint of the E2/E4 ORF with 6 nucleotides deletion. A total of 131 hybrid reads between HPV16 and the cell genome were found, corresponding exactly to the same locus of integration of viral DNA at the 4q25 site. The 178,808 bp genomic deletion was also found in the lingual tumor. We hypothesized that the absence of HPV53 in the lingual tumor was related to the fact that this genotype was associated with the intra-epithelial neoplasia found in the vicinity of the anal carcinoma, and was not involved in the tumor spread. To confirm this hypothesis, we performed macro-dissection of this intra-epithelial neoplasia and DNA extracted was analyzed using CaptHPV method. This analysis found episomal HPV53 DNA sequences, confirming thus the co-occurrence HPV53-associated intra-epithelial lesion and HPV16-associated invasive carcinoma in the anal tissue specimen.

On the whole, the perfect identity of the molecular features of the HPV16 DNA sequences and integration pattern in the two tumors demonstrates that the tongue tumor is a metastasis of the anal carcinoma.

## Conclusion

Using the innovative CaptHPV assay, we report here detailed cartographies of HPV DNA patterns, encompassing full viral sequence, integration signature, integration locus and genomic changes at the insertion site, established from anal and lingual SCCs sequentially developed in the same patient. We show that the identity of these cartographies in the two tumors unambiguously demonstrates that the lingual tumor derived from the anal carcinoma, whereas neither histological immunophenotyping nor classical viral analysis using PCR could allow a definitive diagnosis.

The identity of viral insertion in primary and metastatic tumours has been reported in some cases of Merkel cell carcinoma associated with the Merkel cell polyomavirus (MCPyV) [16], but the use of HPV insertional signature as a tumor marker in the biological follow-up of patients with HPV-associated invasive carcinoma has not been widely developed to date. This is most likely because the DIPS-PCR [17] and APOT [18] methods, commonly used to determine integration locus, provide incomplete results and remain non conclusive in a substantial proportion of cases [8]. NGS-based technology, recently developed in the field of HPV-associated tumors, dramatically modifies the approach by providing, in a single experiment, exhaustive data on the HPV insertional signature [15, 19, 20]. This should favor the development of clinical applications through the identification of personalized tumor markers, useful for diagnostic, predictive, or follow-up purposes [21].

As illustrated in the present observation, the cartography of HPV insertion may be crucial to make the differential diagnosis between metastatic spread and occurrence of an independent tumor, with important consequences in the choice of therapeutic approaches. Diagnostic applications of the NGS-based characterization of HPV-associated tumors should multiply, in particular through the use of the method for the identification of circulating tumor DNA. Such an approach will highly facilitate the diagnosis of relapse when the suspected site of relapse is difficult to access for classical cytological or histological procedure. In such situations, the identification in the blood of the insertion signature, previously determined in the tumor, will constitute a highly specific surrogate for triggering treatment. In a pilot study, we have shown that the mutational insertion could be detected in the blood of cervical cancer patients with equivalent sensitivity as that observed for the detection of only viral sequences [22]. More recently, we have also shown that the NGS-based CaptHPV assay was able to detect this signature in the blood of patients [15]. Besides diagnostic purpose, the detection of circulating HPV DNA is currently in development for use as predictive biomarkers [13, 14] and for the monitoring of immunotherapy [23]. In these perspectives, the detailed cartography of HPV features at the time of diagnosis will permit to choose optimized biomarkers for patients’ serological follow-up.

In certain situations, even the NGS-based approach can be of limited impact to make the differential diagnosis between metastasis and second primary tumors. It is particularly the case when only episomal HPV copies are detected, a situation encountered in 25–30% of cervical cancers [15]. In these cases, the possibility of contamination between samples are discarded taking into account the full viral sequence (presence of variants), the number of reads and their repartition all over the genome. Furthermore, the presence of strictly identical free viral genomes, including variants, in the two tumors does not allow to conclude to the growth of a metastasis, since a same viral genotype can be associated with the development of two independent cell proliferations. The simultaneous characterization of HPV integration pattern and of somatic events should be necessary to obtain definitive diagnosis [24]. NGS-based analysis can also identify cases with highly complex HPV insertion patterns, corresponding to the presence of several integration sites, either clustered or scattered on the cell genome. Changes of these unstable rearrangements may occur over time and lead to the loss the initial site or novel integration sites may develop, thus limiting the identification of the highly specific biomarkers to be used in clinical practice.

The HPV insertion at the 4q25 locus detected in our observation had not been reported so far. *FAM241A* is a potential target gene at this site, deleted through integration in our case of anal carcinoma. No data are available on the potential implication of this gene in oncogenesis. The HPV integration loci were found largely dispersed in the genome but hotspots have been described, among which target genes such as *MYC*, *KLF5/KLF12*, *TP63, VMP1*, *NFIX* [25, 26] have been identified. Our observation was also characterized by the fact that the anal tumor contained two HPV genotypes, HPV53 genomes as free episomes, and HPV16 sequences integrated into the cell genome. Only HPV16 sequences were present in the lingual tumor. Review of the histology of the anal specimen noted the presence of condyloma-like alterations of the epithelium at the vicinity of the invasive carcinoma. It is likely that this corresponded to the presence of a co-infection of the anal mucosa related to HPV53. HPV53, found in 1/505 invasive carcinomas in the French population [1] and in 1.6% (34/2283) of liquid-PAP samples [27], a minority of them (4/34) corresponding to cytological high grade SIL. No progression to malignancy was observed. This genotype is thus considered to have very low oncogenic potential.

On the whole, our observation indicates that the establishment of a detailed cartography of HPV DNA sequences present in a tumor specimen provides crucial information for the design of specific biomarkers that can be used for diagnostic, prognostic or predictive purposes during the patient’s follow-up.

## Abbreviations

APOT: amplification of papillomavirus oncogene transcripts
DIPS-PCR: ligation mediated PCR assay for the detection of integrated papillomavirus sequences
HPV: human papillomavirus
NGS: Next-Generation Sequencing
PCR: polymerase chain reaction
SCC: squamous cell carcinoma
